# Exercise addiction, body dysmorphic disorder, and use of enhancement drugs during the COVID-19 pandemic confinement period: A transcultural study

**DOI:** 10.1192/j.eurpsy.2021.779

**Published:** 2021-08-13

**Authors:** A.R. Dores, I. Carvalho, J. Burkauskas, V. Beretta, K. Ioannidis, P. Simonato, A. Gomez, Z. Demetrovics, H. Fujiwara, S. Chamberlain, N. Fineberg, F. Barbosa, O. Corazza

**Affiliations:** 1 Center For Rehabilitation Research (ess-p.porto), School of Health, Polytechnic of Porto, Porto, Portugal; 2 Laboratory Of Neuropsychophysiology (fpceup); Center For Rehabilitation Research (ess-p.porto), Faculty of Psychology and Education Sciences; School of Health, Polytechnic of Porto, Porto, Portugal; 3 Clinical Neurosciences And Mental Health And Cintesis, School of Medicine, University of Porto,, Porto, Portugal; 4 Neuromokslų Instituto Palangos Klinika, Neuromokslų Instituto Palangos Klinika, Palanga, Lithuania; 5 Department Of Psychiatry, University of Cambridge,, -, United Kingdom; 6 Parco Dei Tigli, Casa di Cura “Parco dei Tigli”, Teolo, Italy; 7 -----, Pontifical University of Salamanca, -, Spain; 8 Institute Of Psychology, ELTE Eotvos Lorand University, Budapest, Hungary; 9 -----, Kyoto University, Japan; 10 Department Of Psychiatry, University of Cambridge, United Kingdom; 11 Laboratory Of Neuropsychophysiology, Faculty Of Psychology And Education Sciences, University of Porto, Porto, Portugal; 12 Department Of Clinical And Pharmaceutical Sciences, University of Hertfordshire, Hatfield, United Kingdom

**Keywords:** COVID-19, Exercise Addiction, body dysmorphic disorder, Use of Enhancement Drugs

## Abstract

**Introduction:**

The Coronavirus pandemic has originated unprecedented sanitary control measures that have conditioned people’s lifestyles and habits. Little is known about the impact of such measures, especially the most restrictive, on recent and growing phenomena such as exercise addiction, use of enhancement drugs, and Body Dysmorphic Disorder (BDD).

**Objectives:**

The objective was to investigate the above-mentioned phenomena during COVID-19 pandemic and how they relate.

**Methods:**

The sample consisted of 3161 participants (65% women), from Portugal (11%), Italy (41%), Spain (16%), the UK (12%), Lithuania (12%), Japan (6%), and Hungary (4%). Mean age was 35.05 (SD = 12.10). Participants responded online to the Exercise Addiction Inventory (EAI), the Appearance Anxiety Inventory (AAI), and questions about use of enhancement drugs.

**Results:**

4.3% of the participants scored above the cut-off point of the EAI, with higher values registered in the UK and Spain. Exercise addiction was higher among men. Appearance anxiety and body satisfaction problems were found in participants of all participating countries, with 15.2% scoring over the cut-off point for BDD. Higher numbers of those at risk of BDD were found in Italy, Japan, and Portugal. About 29% reported the use of fitness supplements to make them look better, with 6.4% starting a new use during the lockdown. Change in supplements use and exercise are predicted by EAI scores. Change in mental health is predicted by AAI scores.

**Conclusions:**

This study helps to shed light on how COVID-19 lookdown induced behavioral changes and how they affect physical and mental health-related aspects in different countries.

